# Changing word meanings in biomedical literature reveal pandemics and new technologies

**DOI:** 10.1186/s13040-023-00332-2

**Published:** 2023-05-05

**Authors:** David N. Nicholson, Faisal Alquaddoomi, Vincent Rubinetti, Casey S. Greene

**Affiliations:** 1 Genomics and Computational Biology Program, University of Pennsylvania, Philadelpia, PA USA; 2 Department of Biomedical Informatics, University of Colorado School of Medicine, Aurora, CO USA; 3 Center for Health Artificial Intelligence (CHAI), University of Colorado School of Medicine, Aurora, CO USA

**Keywords:** Linguistic shift, Pandemic, Software, Novelty

## Abstract

While we often think of words as having a fixed meaning that we use to describe a changing world, words are also dynamic and changing. Scientific research can also be remarkably fast-moving, with new concepts or approaches rapidly gaining mind share. We examined scientific writing, both preprint and pre-publication peer-reviewed text, to identify terms that have changed and examine their use. One particular challenge that we faced was that the shift from closed to open access publishing meant that the size of available corpora changed by over an order of magnitude in the last two decades. We developed an approach to evaluate semantic shift by accounting for both intra- and inter-year variability using multiple integrated models. This analysis revealed thousands of change points in both corpora, including for terms such as ‘cas9’, ‘pandemic’, and ‘sars’. We found that the consistent change-points between pre-publication peer-reviewed and preprinted text are largely related to the COVID-19 pandemic. We also created a web app for exploration that allows users to investigate individual terms (https://greenelab.github.io/word-lapse/). To our knowledge, our research is the first to examine semantic shift in biomedical preprints and pre-publication peer-reviewed text, and provides a foundation for future work to understand how terms acquire new meanings and how peer review affects this process.

**Supplementary Information:**

The online version contains supplementary material available at 10.1186/s13040-023-00332-2.

## Introduction

The meaning of words is constantly evolving. For instance, the word “nice” used to mean foolish or innocent in the fifteenth-seventeenth centuries, before it underwent a shift to its modern meaning of “pleasant or delightful” [1]. This change can be attributed to writers using new metaphors or substituting words with similar meanings, a process known as metonymy [1]. By studying these shifts, we can gain a more nuanced understanding of how language adapts to describe our world.

Scientific fields of inquiry are constantly evolving as researchers develop and test new hypotheses and applications. For example, in the interval studied the CRISPR-Cas9 system has been repurposed as a tool for genome editing. Microbes use this system as a defense against viruses, and scientists have adapted it for genome editing [2], resulting in changes in the use of the term. Written communication is an important part of science [3], both through published papers [4] and preprints [5, 6]. By using computational linguistics to analyze scientific manuscripts, we can identify longitudinal trends in scientific research.

The task of detecting changes in the meaning of words is known as semantic shift detection. This process involves capturing word usage patterns, such as frequency and structure, over a set period of time [7]. Once captured, the final step is generating a time series to show potential shift events, commonly called changepoints [7–9]. By using this approach, researchers have identified many changepoints within publicly available English corpora [10–14]. These discoveries included semantic changes like the meaning of awful shifting from majestic to horrible [15]. In addition to individual discoveries, scientists have identified global patterns that semantic shifts follow [15, 16]. For instance, words with similar meanings, i.e., synonyms, tend to change over time and undergo similar changes [16]. Other patterns include that words change meaning inversely proportional to their frequency, and words with multiple meanings have higher rates of change [15]. Most of these discoveries have been made in regular English text. However, researchers have also attempted to investigate whether these patterns are also found in biomedical literature [17]. The only strong evidence they found is that words that change meaning do so inversely proportional to their usage frequency [18]. Despite conflicting evidence, it is clear that biomedical words and concepts change over time.

Recent studies have investigated semantic shifts in various non-biomedical corpora, such as newspapers [19–21], books [15], Reddit [22], and Twitter [23]. Other research has focused on semantic shifts in topics related to information retrieval [24], and the COVID-19 pandemic has been studied multiple times [25–27]. Additionally, researchers have examined how term usage related to drugs and diseases changes over time [18]. However, with the dramatic increase in open-access biomedical literature over the last two decades, there is an opportunity to analyze semantic shifts in biomedicine on a whole-literature scale. This paper takes a deeper dive into this area by exploring semantic shifts in published and preprint works using natural language-processing and machine learning techniques.

We sought to identify semantic shifts in the rapidly growing body of open-access texts, published papers, and preprints. To do this, we used a novel approach that integrates multiple models to account for the instability of machine learning models trained across various years. This approach allowed us to identify changepoints for each token and to examine key cases. We have made our research products, including changepoints and machine learning models, freely available as open licensed tools for the community. In addition, we have created a web server that allows users to analyze tokens of interest and to observe the most similar terms within a year and temporal trends.

## Methods

### Biomedical corpora examined

#### Pubtator central

Pubtator Central is an open-access resource containing annotated abstracts and full-texts with entity recognition systems for biomedical concepts [28]. The systens used were TaggerOne [29] to tag diseases, chemicals, and cell line entities, GNormPlus [30] to tag genes, SR4GN [31] to tag species, and tmVar [32] to tag genetic mutations. We initially downloaded this resource on December 07th, 2021 and processed over 30 million documents. This resource contains documents from the pre-1800s to 2021; however, due to the low sample size in the early years, we only used documents published from 2000 to 2021. The resource was subsequently updated with documents from 2021. We also downloaded a later version on March 09th, 2022 and merged both versions using each document’s doc_id field to produce the corpus used in this analysis. We divided documents by publication year and then preprocessed each using spacy’s en_core_web_sm model [33]. We replaced each tagged word or phrase with its corresponding entity type and entity ID for every sentence that contained an annotation. Then, we used spacy to break sentences into individual tokens and normalized each token to its root form via lemmatization. After preprocessing, we used every sentence to train multiple Natural Language Processing (NLP) models designed to represent words based on their context.

#### Biomedical preprints

We downloaded a snapshot of BioRxiv [5] and MedRxiv [6] on March 4th, 2022, using their respective Amazon S3 buckets [34, 35]. This snapshot contained 172,868 BioRxiv and 37,517 MedRxiv preprints. We filtered each preprint to its most recent version to prevent duplication bias and sorted them into their respective posted year. Unlike Pubtator Central, these filtered preprints did not contain any annotations. Therefore, we used TaggerOne [29] to tag chemical and disease entities, and GNormplus [30] to tag gene and species entities for our preprint set. We then used spacy to preprocess every preprint as described in the Pubtator Central section.

### Constructing word embeddings for semantic change detection

**Fig. 1:**
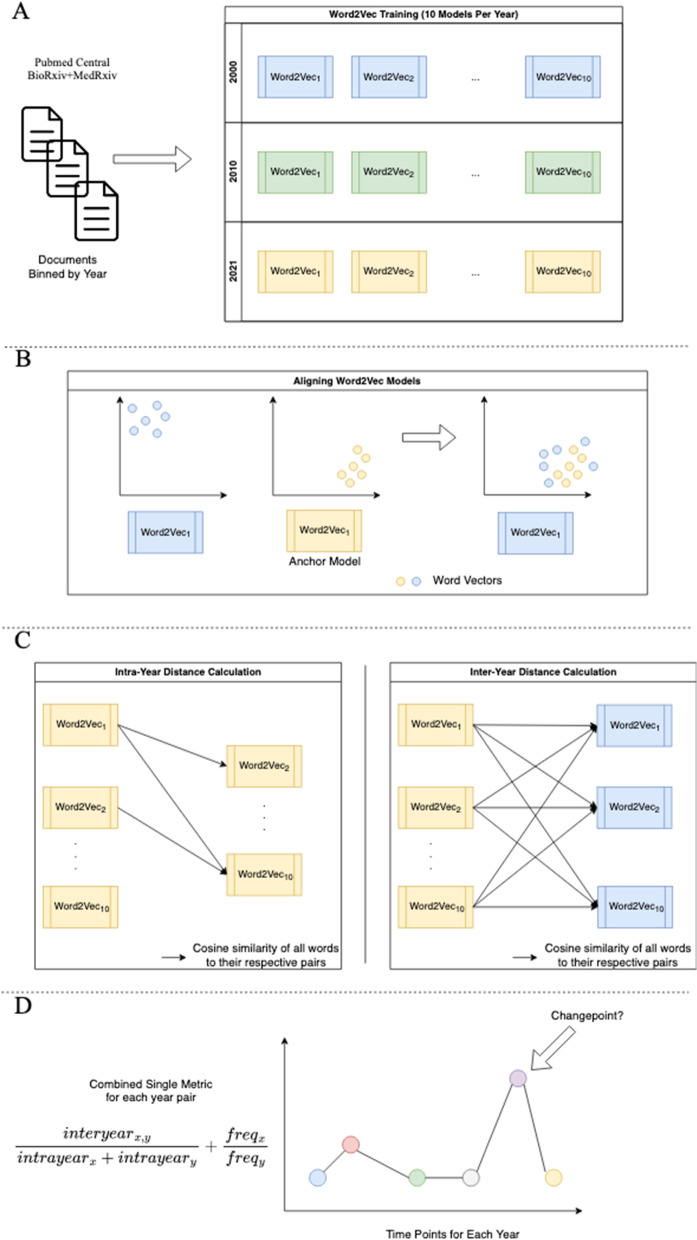
**A** The first step of our data pipeline is where PMCOA papers and BioRxiv/MedRxiv preprints are binned by their respective posting year. Following the binning process, we train ten word2vec models for each year’s manuscripts. **B** Upon training each individual word2vec model, we align every model onto an anchor model. **C** We capture token differences using an intra-year and inter-year approach. Each arrow indicates comparing all tokens from one model with their respective selves in a different model. **D** The last step combines the above calculations into a single metric to allow for a time series to be constructed. Once constructed, we use a statistical technique to autodetect the presence of a changepoint

We used the Word2vec model [36] to construct word vectors for each year. This model is a natural language processing model designed to represent words based on their respective neighbors as dense vectors. The skipgram model generates these vectors by having a shallow neural network predict a word’s neighbors given the word, while the CBOW model predicts the word given its neighbors. We used the CBOW model to construct word vectors for each year. Despite the power of these word2vec models, these models are known to differ due to randomization within a year and year-to-year variability across years [37–40]. To control for run-to-run variability, we examined both intra-year and inter-year relationships. We trained ten different CBOW models for each year using the following parameters: vector size of 300, 10 epochs, minimum frequency cutoff of 5, and a window size of 16 for abstracts (Fig. 1A). Every model has its own unique vector space following training, making it difficult to compare two models without a correction step. We then used orthogonal Procrustes [41] to align all trained CBOW models for the Pubtator Central dataset to the first model trained in 2021, and all CBOW models for the BioRxiv/MedRxiv dataset to the first model trained in 2021 (Fig. 1B). To visualize the aligned models, we used UMAP [42] with the cosine distance metric, a random_state of 100, 25 for n_neighbors, a minimum distance of 0.99, and 50 n_epochs.

### Detecting semantic changes across time

Once the word2vec models were aligned, the next step was to detect semantic change. Semantic change events were detected through time series analysis [10]. We constructed a time series sequence for each token by calculating its distance within a given year (intra-year) and across each year (inter-year) (Fig. 1C). We used the model pairs constructed from the same year to calculate an intra-year distance, which was the cosine distance between each token and its corresponding counterpart. The cosine distance is a metric bounded between 0 and 2, where a score of 0 indicates that two vectors are the same, and a score of 2 indicates that the two vectors are different. For the inter-year distance, we used the Cartesian product of every model between two years and calculated the distance between tokens in the same way as the the intra-year distance. We then combined both metrics by taking the ratio of the average inter-year distance over the average intra-year distance. This approach penalizes tokens with high intra-year instability and rewards more stable tokens. Additionally, it has been shown that including token frequency improves results compared to using distance alone [43]. We calculated token frequency as the ratio of token frequency in the more recent year over the frequency of the previous year. Finally, we combined the frequency with the distance ratios to make the final metric (Fig. 1D).

Following time series construction, we performed change point detection, which uses statistical techniques to detect abnormalities within a given time series (Fig. 1D). We used the CUSUM algorithm [9], which uses a rolling sum of the differences between two timepoints and checks whether the sum is greater than a threshold. A change point is considered to have occurred if the sum exceeds a threshold. We used the 99th percentile on every generated timepoint as the threshold, and ran the CUSUM algorithm with a drift of 0 and default settings for all other parameters.

## Results

### Models can be aligned and compared within and between years

We examined how the usage of tokens in biomedical text changes over time using machine learning models. We trained the models to predict the actual token given a portion of its surrounding tokens, and each token was represented as a vector in a coordinate space constructed by the models.

**Fig. 2:**
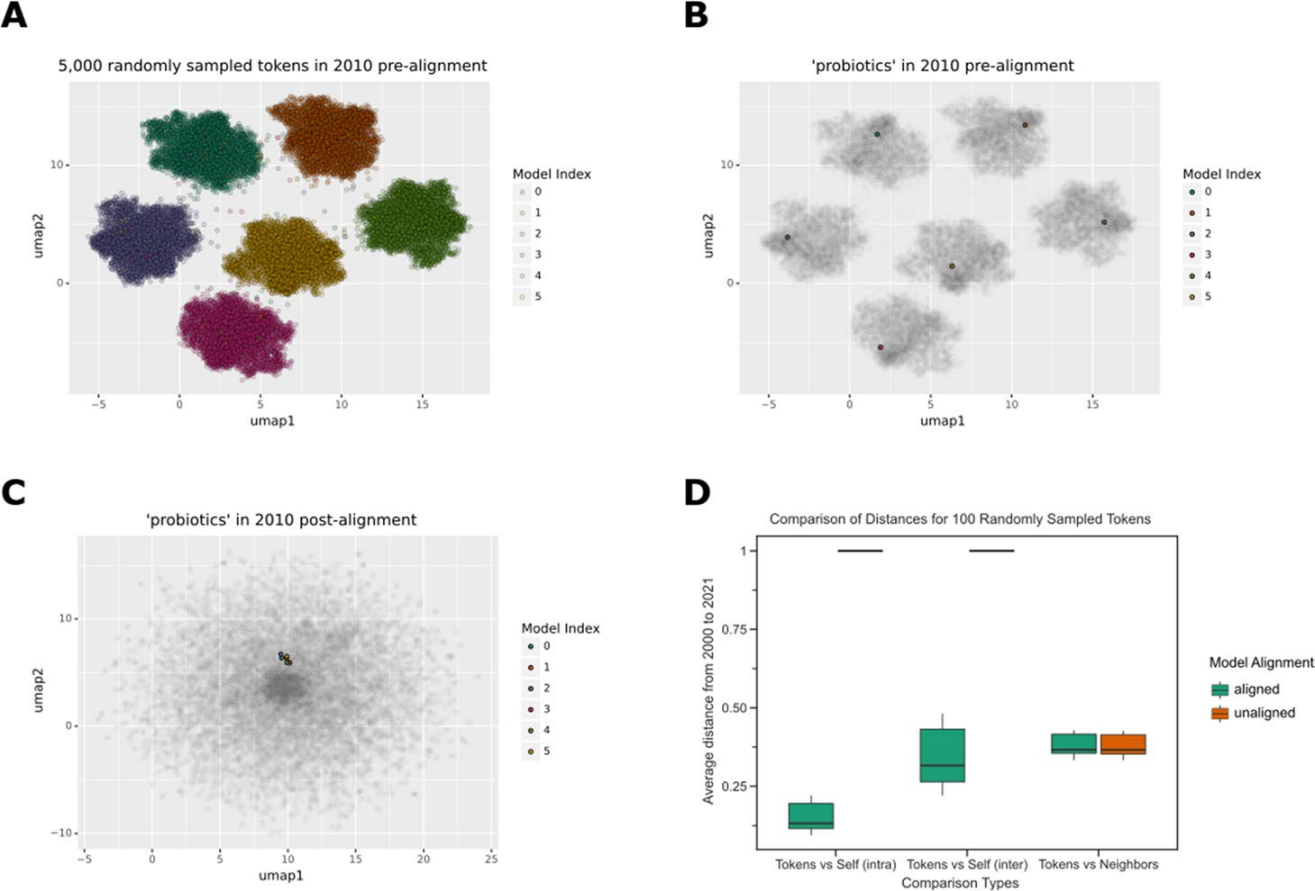
**A** Without alignment, each word2vec model has its own coordinate space. This is a UMAP visualization of 5000 randomly sampled tokens from 5 distinct Word2Vec models trained on the text published in 2010. Each data point represents a token, and the color represents the respective Word2Vec model. **B** We greyed out all tokens except for the token ‘probiotics’ to highlight that each token appears in its own respective cluster without alignment. **C** After the alignment step, the token ‘probiotics’ is closer in vector space signifying that tokens can be easily compared. **D** In the global coordinate space, token distances appear to be vastly different when alignment is not applied. After alignment, token distances become closer; tokens maintain similar distances with their neighbors regardless of alignment. This boxplot shows the average distance of 100 randomly sampled tokens shared in every year from 2000 to 2021. The x-axis shows the various groups being compared (tokens against themselves via intra-year and inter-year distances and tokens against their corresponding neighbors. The y-axis shows the average distance for every year

However, training these models is stochastic, resulting in arbitrary coordinate spaces. Each model has its own unique coordinate space (Fig. 2A), and each word is represented within that space (Fig. 2B). Model alignment is essential in allowing word2vec models to be compared [44, 45]. Alignment projects every model onto a shared coordinate space (Fig. 2C), enabling direct token comparison. To enable comparison of the models, we aligned them onto a shared coordinate space. We randomly selected 100 tokens to confirm that alignment worked as expected. We found that tokens in the global space were more similar to themselves within the year than between years, while identical tokens in unaligned models were completely distinct (Fig. 2D). Local distances were unaffected by alignment, as token-neighbor distances remained unchanged (Fig. 2D).

**Fig. 3:**
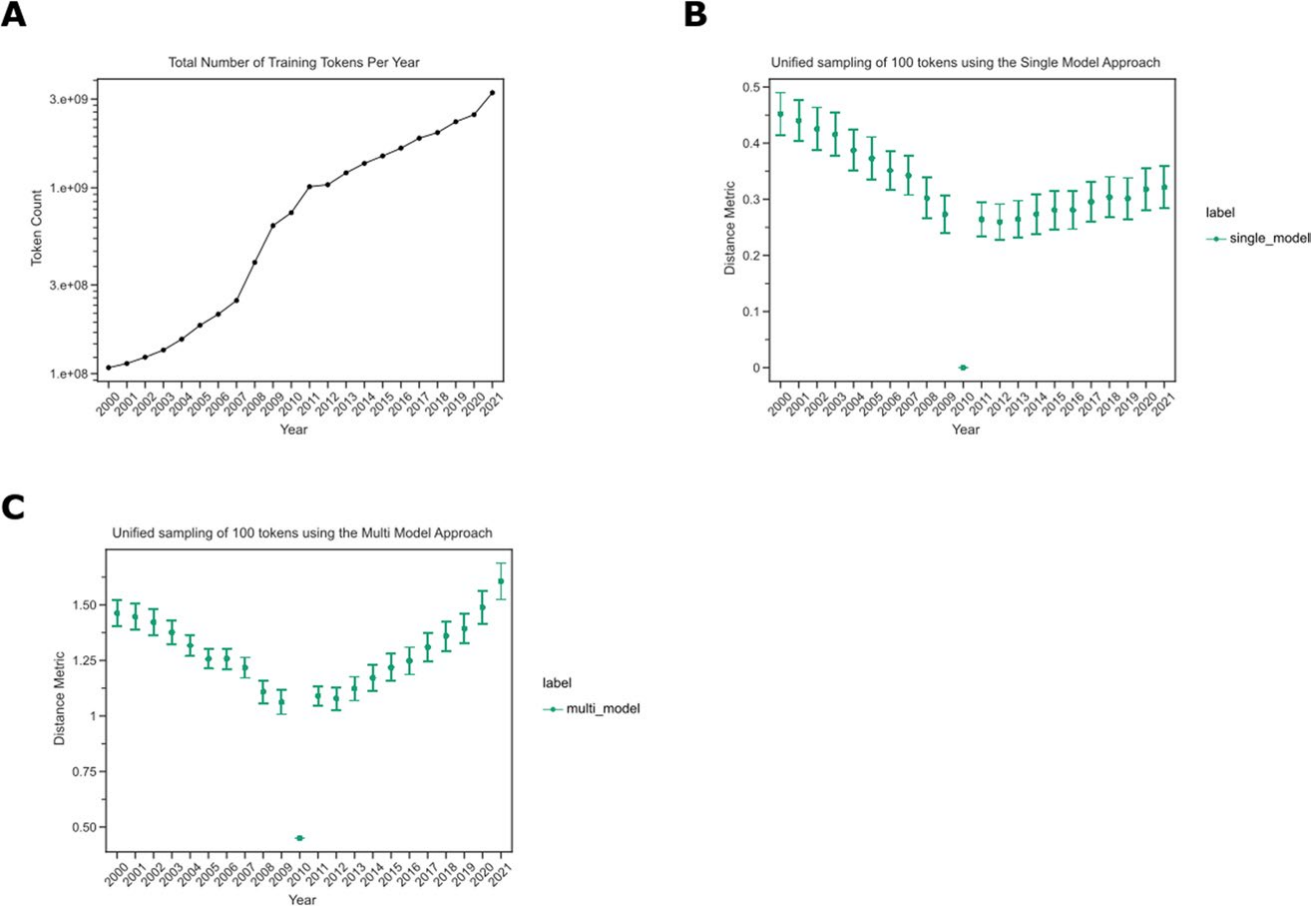
**A** The number of tokens our models have trained on increases over time. This line plot shows the number of unique tokens our various machine-learning models see. The x-axis depicts the year, and the y-axis shows the token count. **B** Earlier years compared to 2010 have greater distances than later years. This confidence interval plot shows the collective distances obtained by sampling 100 tokens present from every year using a single model approach. The x-axis shows a given year, and the y-axis shows the distance metric. **C** Later years have a lower intra-distance variability compared to the earlier years. This confidence interval plot shows the collective distances obtained by sampling 100 tokens present from every year using our multi-model approach. The x-axis shows a given year, and the y-axis shows the distance metric

The landscape of biomedical publishing has changed rapidly during the period of our dataset. The texts for our analysis were open-access manuscripts available through PubMed Central. The growth in the amount of available text and the uneven adoption of open-access publishing during the interval studied was expected to induce changes in the underlying machine learning models, making comparisons more difficult. We found that the number of tokens available for model building, i.e., those in PMC OA, increased dramatically during this time (Fig. 3A). This was expected to create a pattern where models trained in earlier years were more variable than those from later years simply due to the limited sample size in early years. To correct for this change in the underlying models, we developed a statistic that compared tokens’ intra- and inter-year variabilities.

We expected most tokens to undergo minor changes from year to year, while substantial changes likely suggested model drift instead of true linguistic change. We measured the extent to which tokens differed from themselves using the standard single-model approach and our integrated statistic. We filtered the token list to only contain tokens present in every year and compared their distance to the midpoint year, 2010, using the single-model and integrated-models strategies. The single-model approach showed that distances were larger in the earliest years than in later years (Fig. 3B). The integrated model approach did not display the same pattern (Fig. 3C). This suggests that training on smaller corpora leads to high variation and that an integrated model strategy is needed [39]. Therefore, we used the integrated-model strategy for the remainder of this work.

### Terms exhibit detectable changes in usage

**Fig. 4:**
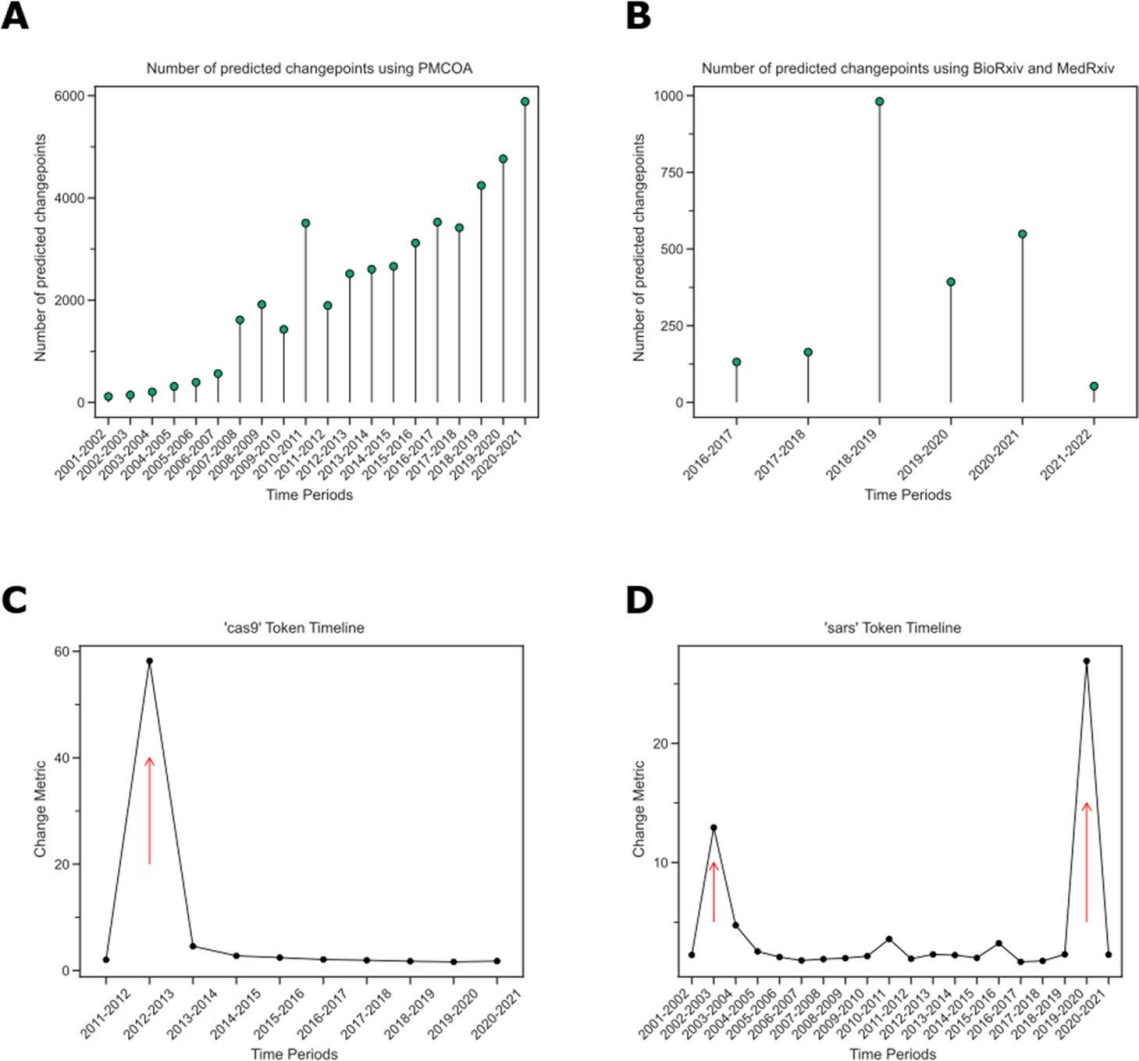
**A** The number of change points increases over time in PMCOA. The x-axis shows the various time periods, while the y-axis depicts the number of detected changepoints. **B** Regarding preprints, the greatest number of change points was during 2018–2019. The x-axis shows the various time periods, while the y-axis depicts the number of detected change points. **C** The token ‘cas9’ was detected to have a changepoint between 2012 and 2013. The x-axis shows the time period since the first appearance of the token, and the y-axis shows the change metric. **D** ‘sars’ has two detected changepoints within the PMCOA corpus. The x-axis shows the time period since the first appearance of the token, and the y-axis shows the change metric

We next sought to identify tokens that changed during the 2000–2021 interval for the text from PubMed Central’s Open Access Corpus (PMCOA) and the 2015–2022 interval for our preprint corpus. We applied the CUSUM algorithm with integrated-model distance to correct for systematic differences in the underlying corpora. We found 41,281 terms with a detected changepoint from PMCOA and 2266 terms from preprints (Fig. 4A and B). Most of our detected changepoints (38,019 for PMCOA and 2260 for preprints) only had a single event.

We detected a changepoint in PMCOA for ‘cas9’ from 2012 to 2013 (Fig. 4C). Before the changepoint, its closest neighbors were related to genetic elements (e.g., ‘cas’1–3). After the changepoint, its closest neighbors became terms related to targeting, sgRNA, gRNA, and other genome editing strategies, such as’talen’ and ‘zfns’ (Table 1). We detected change points for ‘SARS’ from 2002 to 2003 and 2019 to 2020 (Fig. 4D), consistent with the emergences of SARS-CoV [46] and SARS-CoV-2 [47, 48] as observed human pathogens. Before each changepoint, the closest neighbors for ‘SARS’ were difficult to synthesize and summarize. After changepoints, the neighbors for ‘SARS’ were consistent with the acronym for Severe Acute Respiratory Syndrome (Tables 2 and 3).

**Table 1:** The fifteen most similar neighbors to the token ‘cas9’ for the years 2012 and 2013

2012	2013
cas2	sgrna
crispr1	talen
cas3	spcas9
cas1	zfns
cas10	grna
crispr3	zfn
tracrrna	dcas9
crispr	nickase
csn1	pcocas9
crispr4	crispr
cas7	sgrnas
cas6e	meganuclease
cas4	tracrrna
cse1	crispri
cas6	crrna

**Table 2:** The fifteen most similar neighbors to the token ‘sars’ for the years 2002 and 2003

2002	2003
qsar	species_227859
herbicidal	mesh_c000657245
antiplasmodial	severe acute respiratory syndrome-related coronavirus (species_694009)
arylpiperazine	unidentified human coronavirus (species_694448)
a]pyridine	SARS1 (gene_6301)
leishmanicidal	ebola virus sp. (species_205488)
naphthyridine	pandemic
indolo[2,1	coronavirus infections (mesh_d018352)
b]quinazoline-6,12	coronavirus
nematocidal	ebola virus (species_1570291)
f]isoxazolo[2,3	severe acute respiratory syndrome (mesh_d045169)
5-(4	paramyxovirus
cholinephosphotransferase	viruse
oxovanadium(iv	drosten
catecholase	virologist

**Table 3:** The fifteen most similar neighbors to the token ‘sars’ for the years 2019 and 2020

2019	2020
g.o	sar
nsp13	mers
40/367	cov
lissodendoryx	sars-1
lutken	severe acute respiratory syndrome-related coronavirus (species_694009)
sarr	coronaviruse
sar	middle east respiratory syndrome-related coronavirus (species_1335626)
ophiura ophiura (species_72673)	cov
verrill	coronavirus infections (mesh_d018352)
hirondelle	mers-
kobelt	covs
azorean	severe acute respiratory syndrome coronavirus 2 (species_2697049)
rusby	severe acute respiratory syndrome (mesh_d045169)
d’orbigny	sarscov
psychropotes longicauda (species_55639)	sarscov-2

We detected 200 tokens with at least one changepoint in each corpus. Only 25 of the 200 terms were detected to have simultaneous changes between the preprint and PMCOA corpora. We examined the overlap of detected change points between preprints and published articles. Many of these 25 were related to the COVID-19 pandemic (Supplementary Table S1). The complete set of detected change points is available for further analysis (see Data Availability and Software).

### The word-lapse application is an online resource for the manual examination of biomedical tokens

**Fig. 5:**
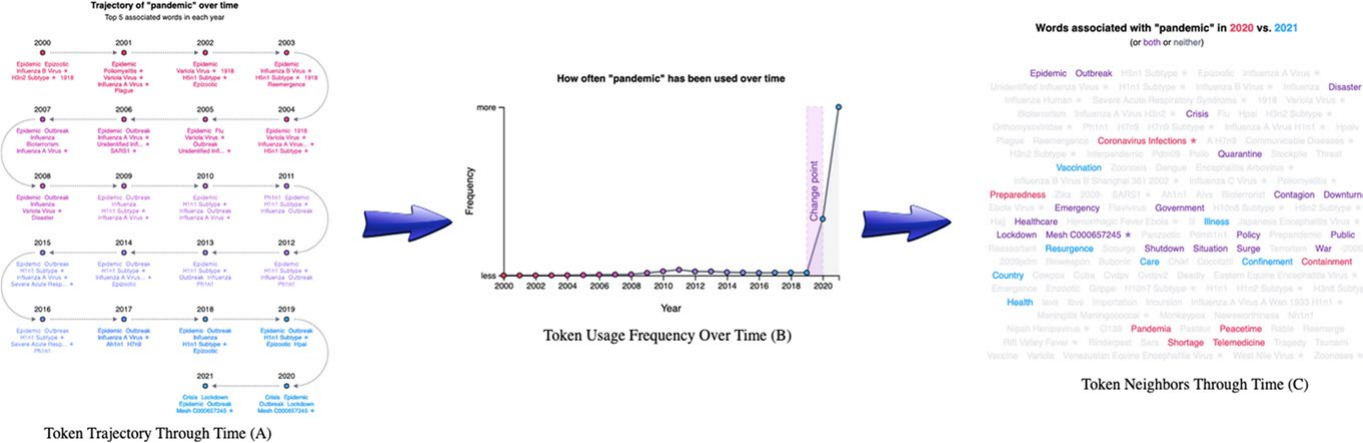
**A** The trajectory visualization of the token ‘pandemic’ through time. It starts at the first mention of the token and progresses through each subsequent year. Every data point shows the top five neighbors for the respective token. **B** The usage frequency of the token ‘pandemic’ through time. The x-axis shows the year, and the y-axis shows the frequency for each token. **C** A word cloud visualization for the top 25 neighbors for the token ‘pandemic’ each year. This visualization highlights each neighbor from a particular year and allows for the comparison between two years. Tokens in purple are shared within both years, while tokens in red or blue are unique to their respective year

Our online application allows users to explore how token meanings change over time. Users can input tokens as text strings, MeSH IDs, Entrez Gene IDs, or Taxonomy IDs. For example, users might elect to explore the term ‘pandemic’, for which we detected a changepoint between 2019 and 2020. The application also shows users the token’s nearest neighbors through time (Fig. 5A). When using ‘pandemic’ as an example, users can observe that ‘epidemic’ remains similar through time, but taxid:114,727 (the H1N1 subtype of influenza) only entered the nearest neighbors with the swine flu pandemic in 2009 and MeSH:C000657245 (COVID-19) appeared in 2020. Additionally, users can view a frequency chart displaying the token’s usage each year (Fig. 5B), which can be displayed as a raw count or adjusted by the total size of the corpus. Previously detected changepoints are indicated on this chart. The final visualization shows the union of the nearest 25 neighbors from each year, ordered by the number of years it was present (Fig. 5C). This visualization includes a comparison function. All functionalities are supported across PMCOA and preprint corpora, and users can toggle between them.

## Discussion

Language is rapidly evolving, and the usage of words changes over time, with words assimilating new meanings or associations [1]. Some efforts have been made to study semantic change using biomedical text [25–27]; however, no such work has examined the changes evident in both pre-publication peer-reviewed and preprinted biomedical text.

We examined semantic changes in two open-access biomedical corpora, PubMed (PMCOA) and bioRxiv/MedRxiv, over a two-decade period from 2000 to 2022. We developed a novel statistic that incorporated multiple Word2Vec models to examine semantic change over time. We used orthogonal procrustes to align each model, and we found that the word vectors were closer together after alignment (Fig. 2). However, the best approach to align these models still remains to be determined [49]. As has been reported in previous studies [39, 50], we found that without a correction step for the variability within and across years, it is difficult to compare stable and unstable models. Our correction approach revealed that the average distances in the earlier years had less variability when using multiple models than when using a single model (Fig. 3).

After correcting for year variability, our analysis revealed more than 41,000 change points, including tokens such as ‘cas9’, ‘pandemic’, and ‘sars’ (Fig. 4). Many of these change points overlapped between PMCOA and preprints, and were related to COVID-19 (Table S1). This indicates that the COVID-19 pandemic has had a sufficiently strong impact on the biomedical literature to cause rapid semantic change across both publishing paradigms [51, 52]. To further investigate these change points, we have developed a web application that allows users to manually examine individual tokens. However, approaches that can automatically validate these change points remain an essential area for future research.

## Conclusion

We uncovered changes in the meanings of words used in biomedical literature using a new approach that took variations between and within years into account. Our approach identified 41,000 changepoints, including well-known terms such as ‘cas9’, ‘pandemic’ and ‘sars’. We created a web application that allows users to investigate these individual changepoints. As a next step, it would be interesting to see if it is possible to detect the consistency and time-lag of semantic changes between preprints and published peer-reviewed texts. This discovery could potentially be used to predict future changes within published texts. Additionally, including other preprint databases may help to uncover consistencies across a wider range of disciplines, or within-field analyses may show the initial stages of semantic changes that will eventually spread throughout biomedicine. Overall, this research is a starting point for understanding semantic changes in biomedical literature, and we are looking forward to seeing how this area develops over time.

## Supplementary Information

**Additional file 1. Table S1.** The intersection of changepoints found between published papers and preprints.

## Data Availability

An online version of this manuscript is available under a Creative Commons Attribution License at https://greenelab.github.io/word_lapse_manuscript/. The source for the research portions of this project is licensed under the BSD-2-Clause Plus Patent at https://github.com/greenelab/biovectors. Our Word Lapse website can be found at https://greenelab.github.io/word-lapse, and the code for the website is available under a BSD-3 Clause at https://github.com/greenelab/word-lapse. Full-text access for the bioRxiv repository is available at https://www.biorxiv.org/tdm. Full-text access for the medRxiv repository is available at https://www.medrxiv.org/tdm. Access to Pubtator Central’s Open Access subset is available on NCBI’s FTP server at https://ftp.ncbi.nlm.nih.gov/pub/lu/PubTatorCentral/.

## References

[1] Semantic Change Elizabeth Closs Traugott Oxford Research Encyclopedia of Linguistics (2017–03–29) DOI: 10.1093/acrefore/9780199384655.013.323. https://doi.org/gp574c

[2] A Programmable Dual-RNA–Guided DNA Endonuclease in Adaptive Bacterial Immunity Martin Jinek, Krzysztof Chylinski, Ines Fonfara, Michael Hauer, Jennifer A Doudna, Emmanuelle Charpentier Science (2012–08–17) DOI: 10.1126/science.1225829. https://doi.org/f22dgd PMID: 22745249 · PMCID: PMC6286148 10.1126/science.1225829 PMC6286148 22745249

[3] Scientific communication pathways: an overview and introduction to a symposium David F Zaye, WV Metanomski Journal of Chemical Information and Computer Sciences (1986–05–01) DOI: 10.1021/ci00050a001. https://doi.org/bwsxhg

[4] PubMed Central: The GenBank of the published literature Richard J Roberts Proceedings of the National Academy of Sciences (2001–01–09) DOI: 10.1073/pnas.98.2.381. https://doi.org/bbn9k8 PMID: 11209037 · PMCID: PMC33354 10.1073/pnas.98.2.381 PMC33354 11209037

[5] bioRxiv: the preprint server for biology Richard Sever, Ted Roeder, Samantha Hindle, Linda Sussman, Kevin-John Black, Janet Argentine, Wayne Manos, John R Inglis Cold Spring Harbor Laboratory (2019–11–06). 10.1101/833400. https://doi.org/ggc46z

[6] Medical preprint server debuts Jocelyn Kaiser Science (2019–06–05). 10.1126/science.aay2933. https://doi.org/gpxkkf

[7] Diachronic word embeddings and semantic shifts: a survey Andrey Kutuzov, Lilja Øvrelid, Terrence Szymanski, Erik Velldal arXiv (2018–06–14) https://arxiv.org/abs/1806.03537

[8] Bayesian Online Changepoint Detection Ryan Prescott Adams, David JC MacKay arXiv (2007–10–22) https://arxiv.org/abs/0710.3742

[9] Adaptive filtering and change detection Fredrik Gustafsson (2000). 10.1002/0470841613.

[10] Statistically Significant Detection of Linguistic Change Vivek Kulkarni, Rami Al-Rfou, Bryan Perozzi, Steven Skiena Proceedings of the 24th International Conference on World Wide Web (2015–05–18) :DOI: 10.1145/2736277.2741627. https://doi.org/ghcv6k

[11] A framework for analyzing semantic change of words across time Adam Jatowt, Kevin Duh IEEE/ACM Joint Conference on Digital Libraries (2014–09) DOI: 10.1109/jcdl.2014.6970173. https://doi.org/gp8zpm

[12] Understanding semantic change of words over centuries Derry Tanti Wijaya, Reyyan Yeniterzi Proceedings of the 2011 international workshop on DETecting and Exploiting Cultural diversiTy on the social web (2011–10–24) DOI: 10.1145/2064448.2064475. https://doi.org/cmxz2v

[13] Deep Neural Models of Semantic Shift Alex Rosenfeld, Katrin Erk Proceedings of the 2018 Conference of the North American Chapter of the Association for Computational Linguistics: Human Language Technologies, Volume 1 (Long Papers) (2018) DOI: 10.18653/v1/n18-1044. https://doi.org/gp574f

[14] A state-of-the-art of semantic change computation XURI TANG Natural Language Engineering (2018–06–18) DOI: 10.1017/s1351324918000220. https://doi.org/gkkswt

[15] Diachronic Word Embeddings Reveal Statistical Laws of Semantic Change William L Hamilton, Jure Leskovec, Dan Jurafsky arXiv (2018–10–26) https://arxiv.org/abs/1605.09096

[16] Yang X, Kemp C. A computational evaluation of two laws of semantic change. CogSci. 2015.

[17] Tracking word semantic change in biomedical literature Erjia Yan, Yongjun Zhu International Journal of Medical Informatics (2018–01) DOI: 10.1016/j.ijmedinf.2017.11.006. https://doi.org/grwsdh · PMID: 29195709 10.1016/j.ijmedinf.2017.11.006 29195709

[18] Exploring Diachronic Changes of Biomedical Knowledge using Distributed Concept Representations Gaurav Vashisth, Jan-Niklas Voigt-Antons, Michael Mikhailov, Roland Roller Proceedings of the 18th BioNLP Workshop and Shared Task (2019) DOI: 10.18653/v1/w19-5037. https://doi.org/grwsdj

[19] Tracing armed conflicts with diachronic word embedding models Andrey Kutuzov, Erik Velldal, Lilja Øvrelid Proceedings of the Events and Stories in the News Workshop (2017) DOI: 10.18653/v1/w17-2705. https://doi.org/ghx5gj

[20] Words are Malleable: Computing Semantic Shifts in Political and Media Discourse Hosein Azarbonyad, Mostafa Dehghani, Kaspar Beelen, Alexandra Arkut, Maarten Marx, Jaap Kamps arXiv (2017–11–16) https://arxiv.org/abs/1711.05603

[21] Reading Between the Lines: Prediction of Political Violence Using Newspaper Text HANNES MUELLER, CHRISTOPHER RAUH American Political Science Review (2017–12–14) DOI: 10.1017/s0003055417000570. https://doi.org/gdj77d

[22] Detection of emerging drugs involved in overdose via diachronic word embeddings of substances discussed on social media Austin P Wright, Christopher M Jones, Duen Horng Chau, R Matthew Gladden, Steven A Sumner Journal of Biomedical Informatics (2021–07) DOI: 10.1016/j.jbi.2021.103824. https://doi.org/gp8zph · PMID: 34048933 10.1016/j.jbi.2021.103824 PMC10901232 34048933

[23] Statistically Significant Detection of Linguistic Change Vivek Kulkarni, Rami Al-Rfou, Bryan Perozzi, Steven Skiena arXiv (2014–11–13) https://arxiv.org/abs/1411.3315

[24] Semantic word shifts in a scientific domain Baitong Chen, Ying Ding, Feicheng Ma Scientometrics (2018–07–13) DOI: 10.1007/s11192-018-2843-2. https://doi.org/gd7bd7

[25] Semantic Changepoint Detection for Finding Potentially Novel Research Publications Bhavish Dinakar, Mayla R Boguslav, Carsten Görg, Deendayal Dinakarpandian Biocomputing 2021 (2020–11) DOI: 10.1142/9789811232701_0011. https://doi.org/gp574d PMC8352552 33691009

[26] How COVID-19 Is Changing Our Language : Detecting Semantic Shift in Twitter Word Embeddings Yanzhu Guo, Christos Xypolopoulos, Michalis Vazirgiannis arXiv (2021–02–17) https://arxiv.org/abs/2102.07836

[27] Natural Language Processing Reveals Vulnerable Mental Health Support Groups and Heightened Health Anxiety on Reddit During COVID-19: Observational Study Daniel M Low, Laurie Rumker, Tanya Talkar, John Torous, Guillermo Cecchi, Satrajit S Ghosh Journal of Medical Internet Research (2020–10–12) DOI: 10.2196/22635. https://doi.org/ghm9v2 · PMID: 32936777 · PMCID: PMC7575341 10.2196/22635 PMC7575341 32936777

[28] PubTator central: automated concept annotation for biomedical full text articles Chih-Hsuan Wei, Alexis Allot, Robert Leaman, Zhiyong Lu Nucleic Acids Research (2019–05–22) DOI: 10.1093/nar/gkz389. https://doi.org/ggzfsc · PMID: 31114887 · PMCID: PMC6602571 10.1093/nar/gkz389 PMC6602571 31114887

[29] TaggerOne: joint named entity recognition and normalization with semi-Markov Models Robert Leaman, Zhiyong Lu Bioinformatics (2016–06–09) DOI: 10.1093/bioinformatics/btw343. https://doi.org/f855dg · PMID: 27283952 · PMCID: PMC5018376 10.1093/bioinformatics/btw343 PMC5018376 27283952

[30] GNormPlus: An Integrative Approach for Tagging Genes, Gene Families, and Protein Domains Chih-Hsuan Wei, Hung-Yu Kao, Zhiyong Lu BioMed Research International (2015) DOI: 10.1155/2015/918710. https://doi.org/gb85jb · PMID: 26380306 · PMCID: PMC4561873 10.1155/2015/918710 PMC4561873 26380306

[31] SR4GN: A Species Recognition Software Tool for Gene Normalization Chih-Hsuan Wei, Hung-Yu Kao, Zhiyong Lu PLoS ONE (2012–06–05) DOI: 10.1371/journal.pone.0038460. https://doi.org/gpq498 · PMID: 22679507 · PMCID: PMC3367953 10.1371/journal.pone.0038460 PMC3367953 22679507

[32] tmVar 2.0: integrating genomic variant information from literature with dbSNP and ClinVar for precision medicine Chih-Hsuan Wei, Lon Phan, Juliana Feltz, Rama Maiti, Tim Hefferon, Zhiyong Lu Bioinformatics (2017-09-01) DOI: 10.1093/bioinformatics/btx541. https://doi.org/gbzsmc · PMID: 28968638 · PMCID: PMC5860583 10.1093/bioinformatics/btx541 PMC5860583 28968638

[33] Honnibal M, Montani I. spaCy 2: Natural language understanding with Bloom embeddings, convolutional neural networks and incremental parsing Matthew. 2017.

[34] Machine access and text/data mining resources | bioRxiv https://www.biorxiv.org/tdm

[35] Machine access and text/data mining resources | medRxiv https://www.medrxiv.org/tdm

[36] Efficient Estimation of Word Representations in Vector Space Tomas Mikolov, Kai Chen, Greg Corrado, Jeffrey Dean arXiv (2013–09–10) https://arxiv.org/abs/1301.3781

[37] Factors Influencing the Surprising Instability of Word Embeddings Laura Wendlandt, Jonathan K Kummerfeld, Rada Mihalcea arXiv (2020–06–05) https://arxiv.org/abs/1804.09692 DOI: 10.18653/v1/n18-1190

[38] Stability of Word Embeddings Using Word2Vec Mansi Chugh, Peter A Whigham, Grant Dick AI 2018: Advances in Artificial Intelligence (2018) DOI: 10.1007/978-3-030-03991-2_73. https://doi.org/gpxkkc

[39] Evaluating the Stability of Embedding-based Word Similarities Maria Antoniak, David Mimno Transactions of the Association for Computational Linguistics (2018–12) DOI: 10.1162/tacl_a_00008. https://doi.org/gf39k8

[40] Predicting Word Embeddings Variability Benedicte Pierrejean, Ludovic Tanguy Proceedings of the Seventh Joint Conference on Lexical and Computational Semantics (2018) DOI: 10.18653/v1/s18-2019. https://doi.org/gh6qpc

[41] A generalized solution of the orthogonal procrustes problem Peter H Schönemann Psychometrika (1966–03) DOI: 10.1007/bf02289451. https://doi.org/dx77sz

[42] UMAP: Uniform Manifold Approximation and Projection for Dimension Reduction Leland McInnes, John Healy, James Melville arXiv (2020–09–21) https://arxiv.org/abs/1802.03426

[43] Improving semantic change analysis by combining word embeddings and word frequencies Adrian Englhardt, Jens Willkomm, Martin Schäler, Klemens Böhm International Journal on Digital Libraries (2019–05–20) DOI: 10.1007/s00799-019-00271-6. https://doi.org/gpxkkd

[44] Diachronic Word Embeddings Reveal Statistical Laws of Semantic Change William L Hamilton, Jure Leskovec, Dan Jurafsky arXiv (2016) DOI: 10.48550/arxiv.1605.09096. https://doi.org/gp8zpp

[45] DUKweb, diachronic word representations from the UK Web Archive corpus Adam Tsakalidis, Pierpaolo Basile, Marya Bazzi, Mihai Cucuringu, Barbara McGillivray Scientific Data (2021–10–15) DOI: 10.1038/s41597-021-01047-x. https://doi.org/gqbkx4 · PMID: 34654827 · PMCID: PMC8520005 10.1038/s41597-021-01047-x PMC8520005 34654827

[46] SARS: clinical virology and pathogenesis John NICHOLLS, Xiao-Ping DONG, Gu JIANG, Malik PEIRIS Respirology (2003–11) DOI: 10.1046/j.1440-1843.2003.00517.x. https://doi.org/cxjwrc · PMID: 15018126 · PMCID: PMC7169081 10.1046/j.1440-1843.2003.00517.x PMC7169081 15018126

[47] Severe acute respiratory syndrome coronavirus 2 (SARS-CoV-2) and coronavirus disease-2019 (COVID-19): The epidemic and the challenges Chih-Cheng Lai, Tzu-Ping Shih, Wen-Chien Ko, Hung-Jen Tang, Po-Ren Hsueh International Journal of Antimicrobial Agents (2020–03) DOI: 10.1016/j.ijantimicag.2020.105924. https://doi.org/ggpj9d · PMID: 32081636 · PMCID: PMC7127800 10.1016/j.ijantimicag.2020.105924 PMC7127800 32081636

[48] The species Severe acute respiratory syndrome-related coronavirus: classifying 2019-nCoV and naming it SARS-CoV-2, Alexander E Gorbalenya, Susan C Baker, Ralph S Baric, Raoul J de Groot, Christian Drosten, Anastasia A Gulyaeva, Bart L Haagmans, Chris Lauber, Andrey M Leontovich, … John Ziebuhr Nature Microbiology (2020–03–02) DOI: 10.1038/s41564-020-0695-z. https://doi.org/ggqj7m · PMID: 32123347 · PMCID: PMC7095448 10.1038/s41564-020-0695-z PMC7095448 32123347

[49] Learning Diachronic Word Embeddings with Iterative Stable Information Alignment Zefeng Lin, Xiaojun Wan, Zongming Guo Natural Language Processing and Chinese Computing (2019) DOI: 10.1007/978-3-030-32233-5_58. https://doi.org/gp8zpg

[50] Factors Influencing the Surprising Instability of Word Embeddings Laura Wendlandt, Jonathan K Kummerfeld, Rada Mihalcea arXiv (2018) DOI: 10.48550/arxiv.1804.09692. https://doi.org/gqcn9m

[51] The evolving role of preprints in the dissemination of COVID-19 research and their impact on the science communication landscape Nicholas Fraser, Liam Brierley, Gautam Dey, Jessica K Polka, Máté Pálfy, Federico Nanni, Jonathon Alexis Coates PLOS Biology (2021–04–02) DOI: 10.1371/journal.pbio.3000959. https://doi.org/gk6s8d · PMID: 33798194 · PMCID: PMC8046348 10.1371/journal.pbio.3000959 PMC8046348 33798194

[52] Characteristics of academic publications, preprints, and registered clinical trials on the COVID-19 pandemic Silvia Gianola, Tiago S Jesus, Silvia Bargeri, Greta Castellini PLOS ONE (2020–10–06) DOI: 10.1371/journal.pone.0240123. https://doi.org/ghgdxw · PMID: 33022014 · PMCID: PMC7537872 10.1371/journal.pone.0240123 PMC7537872 33022014

[53] A publishing infrastructure for AI-assisted academic authoring Milton Pividori, Casey S Greene Cold Spring Harbor Laboratory (2023–01–23) DOI: 10.1101/2023.01.21.525030. https://doi.org/grpf8m · PMID: 36747665 · PMCID: PMC9900745

