# Supplementary material for: Changing word meanings in biomedical literature reveal pandemics and new technologies
Source: BioData Min. 2023 May 5;16:16. doi: 10.1186/s13040-023-00332-2 (PMC10161184; doi:10.1186/s13040-023-00332-2)
Supplement: Supplementary file 1 — Additional file 1. Table S1. The intersection of changepoints found between published papers and preprints. [file 13040_2023_332_MOESM1_ESM.docx]

## Supplemental Tables

Table S1: The intersection of changepoints found between published papers and preprints.

| Token | Changepoint |
| --- | --- |
| lockdown | 2019-2020 |
| 2021 | 2020-2021 |
| distancing | 2019-2020 |
| 2019 | 2018-2019 |
| ace2 | 2019-2020 |
| pandemic | 2019-2020 |
| 2020 | 2019-2020 |
| coronavirus | 2019-2020 |
| bcl2a1 | 2018-2019 |
| peak3 | 2020-2021 |
| 3.6.2 | 2019-2020 |
| quarantine | 2019-2020 |
| cobl | 2020-2021 |
| injectrode | 2020-2021 |
| nrc3 | 2020-2021 |
| 4.0.5 | 2020-2021 |
| TMPRSS2 (gene_7113) | 2019-2020 |
| n262 | 2019-2020 |
| bin1 | 2017-2018 |
| n3c | 2020-2021 |
| tip1 | 2020-2021 |
| omicron | 2020-2021 |
| pangolin | 2019-2020 |
| adrn | 2020-2021 |
| seir | 2019-2020 |

# 
